# Statistical analysis plan for the Stroke Oxygen Study (SO_2_S): a multi-center randomized controlled trial to assess whether routine oxygen supplementation in the first 72 hours after a stroke improves long-term outcome

**DOI:** 10.1186/1745-6215-15-229

**Published:** 2014-06-16

**Authors:** Julius Sim, Richard Gray, Tracy Nevatte, Andrew Howman, Natalie Ives, Christine Roffe

**Affiliations:** 1 Health Services Research Unit, Keele University, Keele ST5 5BG, UK; 2 Clinical Trial Service Unit, University of Oxford, Roosevelt Drive, Oxford OX3 7LF, UK; 3 Stroke Research, Institute for Science and Technology in Medicine, Keele University, Keele ST5 5BG, UK; 4 Birmingham Clinical Trials Unit, Robert Aitken Institute, University of Birmingham, Birmingham B15 2TT, UK; 5 Stroke Research, North Staffordshire Combined Healthcare NHS Trust, Holly Lodge, 62 Queens Road, Stoke on Trent, Staffordshire ST4 7LH, UK

**Keywords:** hypoxia, oxygen supplementation, randomized controlled trial, statistical analysis plan, stroke

## Abstract

**Background:**

The Stroke Oxygen Study (SO_2_S) is a multi-center randomized controlled trial of oxygen supplementation in patients with acute stroke. The main hypothesis for the trial is that fixed-dose oxygen treatment during the first 3 days after an acute stroke improves outcome. The secondary hypothesis is that restricting oxygen supplementation to night time only is more effective than continuous supplementation. This paper describes the statistical analysis plan for the study.

**Methods and design:**

Patients (*n* = 8000) are randomized to three groups: (1) continuous oxygen supplementation for 72 hours; (2) nocturnal oxygen supplementation for three nights; and (3) no routine oxygen supplementation. Outcomes are recorded at 7 days, 90 days, 6 months, and 12 months. The primary outcome measure is the modified Rankin scale at 90 days. Data will be analyzed according to the intention-to-treat principle. Methods of statistical analysis are described, including the handling of missing data, the covariates used in adjusted analyses, planned subgroups analyses, and planned sensitivity analyses.

**Trial registration:**

This trial is registered with the ISRCTN register, number ISRCTN52416964 (30 September 2005).

## Background

Whilst it is generally acknowledged that specialist care on stroke units is effective in preventing death and disability after stroke [1], it is not known which aspects of stroke care are crucial in this respect. Mild hypoxia commonly occurs in stroke patients and hypoxemia in the first few hours after admission to hospital admission is associated with an increased risk of death [2]. Guidance for oxygen treatment after stroke differs between countries, and is not based on evidence from randomized controlled trials. In many accident and emergency departments in the UK, oxygen is given routinely to stroke patients regardless of blood oxygen levels. In support of the use of oxygen is evidence that eubaric hyperoxia may be neuroprotective [3,4]. However, there is also evidence that oxygen encourages the formation of toxic free radicals, leading to further damage to the ischemic brain [5-8]. Additionally, the tubing used to deliver oxygen impedes mobilization and may pose a risk of infection. A study of eubaric hyperoxia using extremely high-flow oxygen supplementation (45 l/min) for 8 hours was abandoned in 2009 because of increased mortality in the oxygen group (Singhal *et al.*, unpublished work: http://www.clinicaltrials.gov).

Patients are more likely to be hypoxic at night [9,10], hypoxia is more likely to be missed at this time, and nocturnal hypoxemia carries a greater risk of brain tissue hypoxia [11]. Nocturnal oxygen supplementation does not interfere with the patient’s daytime mobility and therefore does not affect early mobilization [12]. Giving routine oxygen only at night might therefore prevent a significant number of otherwise undetected episodes of hypoxia without interfering with the patient’s daytime rehabilitation.

The Stroke Oxygen Study (SO_2_S) evaluated the use of low-dose oxygen therapy in the first 72 hours following stroke [13]. The aim of this study is, firstly, to assess whether patients benefit from oxygen after stroke, and secondly to establish whether nocturnal oxygen supplementation is more effective than continuous oxygen supplementation. Fuller details of the rationale and the design the study are given in the study protocol; this paper describes the statistical analyses to be undertaken on the study data. It should be read in conjunction with the protocol [13].

## Study design

The Stroke Oxygen Study is a multi-center randomized controlled trial of oxygen supplementation in patients with acute stroke.

Patients are randomized in a ratio of 1:1:1 to three groups: (1) continuous oxygen supplementation for 72 hours; (2) nocturnal oxygen supplementation for 3 nights; and (3) no routine oxygen supplementation. Oxygen is given at a rate of 2 to 3 l/min, depending on baseline oxygen saturation. Randomization is conducted via a web-based interface using minimization. The minimization variables are: (1) the ‘six simple variables’ (SSV) score for being alive and independent at 6 months (derived from age, and yes/no scores for: living alone, pre-stroke independence in activities of daily living, normal speech, ability to lift both arms, ability to walk) [14,15], with the cut-offs <0.1, >0.1 to ≤0.35, >0.35 to ≤0.70, and >0.70; (2) oxygen given in the ambulance or hospital prior to randomization (yes, no, or unknown); (3) oxygen saturation on room air at randomization (<95 and ≥95%); and (4) time since stroke onset (≤3, >3 to ≤6, >6 to ≤12, >12 to ≤24 and >24 hours). Patients are followed up at 7 days, 90 days, 180 days, and 1 year [13].

Multi-center ethical approval was granted for SO_2_S by the North Staffordshire Research Ethics Committee on 25 June 2008 (COREC 06/Q2604/109). Informed consent was obtained from each participant in the study [13].

### Main hypothesis

Fixed-dose oxygen treatment during the first 3 days after an acute stroke improves outcome.

### Secondary hypothesis

Restricting oxygen supplementation to night time only is more effective than continuous supplementation.

## Sample size

The original sample size for the study was 6,000 participants. This would detect an odds ratio of 0.83 for a one-point difference in the modified Rankin scale (mRS) score in a comparison of the combined oxygen supplementation groups and the control group, assuming 95% power and a 5% two-tailed significance level. With allowance for 10% loss to follow-up, this gave a recruitment target of 6,669. The recruitment target was subsequently revised in October 2012 to 8,000 patients, to provide greater power to detect an interaction between subgroups (defined by severity) and the effect of oxygen versus control; see the study protocol [13].

## General considerations

### Levels of confidence and *P* values

Unless otherwise specified, estimates of treatment effects will be presented with 95% confidence intervals. A two-tailed *P* value of ≤0.05 will be considered statistically significant for the primary outcome measure. For the analysis of secondary outcomes, a two-tailed *P* value of ≤0.01 will be considered statistically significant and estimates will be presented with 99% confidence intervals.

### Protocol violations and exclusions from the study

Analysis will be according to the intention-to-treat principle, meaning that all patients will be analyzed in the treatment arm to which they were randomized, and all patients will be included, whether or not they received the allocated treatment. Protocol deviations and exclusions (and reasons for any such exclusions) will be reported for each arm of the trial.

### Unadjusted and adjusted analyses

For each outcome variable, the unadjusted analysis will be designated the primary analysis; the covariate-adjusted analysis will be designated the secondary analysis. Adjusted analyses will incorporate the following covariates: age, sex, baseline National Institutes of Health Stroke Scale (NIHSS) score [16], baseline oxygen saturation, the SSV prognostic index for 6-month independence (for analysis of mortality data, the prognostic index for 30-day survival will be used in place of that for six-month independence).

### Missing data

Every effort will be made to obtain missing data, even after the follow-up time has passed. Patients who are contactable at 6 months, but have not completed the 3-month questionnaire, will be asked to think back to 3 months and consider for each missing data point what they were like at that time. Where it is impossible to get any information from the patients or friends or family members designated by the patients as alternative contacts, we will contact the patients’ general practitioner or the investigators at the hospital where they were admitted to determine whether there is information on record (such as discharge letters or clinic letters that provide information on the patient’s status at or near the time of the missing assessment). Some of the information on the questionnaires is duplicated; that is, items measuring a particular variable exist in more than one questionnaire. Where such information is provided in one questionnaire but omitted in another, the relevant data will be used to complete the missing data, where possible.

Missing data that remain following such efforts will be estimated through multiple imputation. The main analyses will be based on available data only (including information secured by the strategies described above). Analyses based on imputed data will be reported as part of the sensitivity analyses (described later).

### Interim analyses and stopping rules

Interim analyses of efficacy and safety for the data safety and monitoring committee are planned annually. These analyses will be considered in the closed session of the committee. The committee will advise the chair of the trial steering committee if, in their view, the randomized comparisons in the trial have provided both (a) ‘proof beyond reasonable doubt’ that for all, or for some types of, patients one particular treatment is definitely indicated or definitely contraindicated in terms of a net difference in the major endpoints, and (b) evidence that might reasonably be expected to influence patient management by many clinicians who are already aware of the other main trial results. Appropriate criteria for proof beyond reasonable doubt are not specified precisely, but an effect equivalent to a difference of at least three standard deviations in an interim analysis of a major endpoint might be seen as a reasonable justification for modifying the protocol or for halting the study prematurely.

### Start of data analysis

The first analysis of the main trial data for publication purposes will begin once the final randomized patient has reached the 90-day follow-up and missing data have been chased. We will aim to achieve at least 95% follow-up rates for the main outcome. The database will be frozen and the statistical analysis plan agreed before any analysis is carried out.

The second analysis of the main trial data for publication purposes will begin once the final randomized patient has reached the 12-month follow-up, using the same procedures. We will again aim for >95% returns for the main outcome, but will accept >90% if this is not achieved within 3 months of the last due response.

## Proposed analyses

### Treatment comparisons

Two distinct treatment comparisons are planned in this study. The first is to compare oxygen supplementation with no oxygen supplementation (with the former group comprising both patients who were allocated nocturnal oxygen only for three nights and patients allocated continuous oxygen for 72 hours). The second comparison compares the two oxygen supplementation groups with each other (nocturnal oxygen only for three nights versus continuous oxygen for 72 hours).

In addition to the two main comparisons, in the event that, in relation to the primary outcome measure, (a) the overall comparison between both oxygen supplementation groups and the control group is non-significant, but (b) one oxygen supplementation group shows significantly greater benefit than the other, a comparison will be performed (on the primary outcome measure only) between the better of the two oxygen supplementation groups and the control group. This is to examine the possibility that, for example, nocturnal oxygen supplementation may have a beneficial effect that is offset by, and thus masked by, a non-beneficial or harmful effect of continuous oxygen supplementation.

The analyses described next will be repeated for each of the two main comparisons.

### Primary outcome measure

The primary outcome measure is the modified Rankin scale (mRS) at 90 days after randomization [17]. The mRS is an ordinal scale ranging from 0 (no disability) to 5 (extreme disability). Patients who die prior to the 90-day time point will be considered to have an mRS score of 6, thus creating a 0 to 6 scale.

The mRS will be analyzed using an ordinal logistic regression model. Both an unadjusted (primary) and adjusted (secondary) analysis will be performed using the covariates specified earlier (see General considerations section).

### Secondary outcome measures at 7 days

Secondary outcome measures at 7 days comprise: the NIHSS; the number of patients with neurological improvement (≥4 point decrease from baseline or a value of 0 in the NIHSS); mortality; highest oxygen saturation during the first 72 hours; lowest oxygen saturation during the first 72 hours; the number of patients whose oxygen saturation falls below 90%.

### Secondary outcome measures at 90 days

Secondary outcome measures at 90 days comprise: mortality; the number of patients alive and independent (mRS ≤2); the number of patients living at home; ability to perform activities of daily using (using the Barthel index of activities of daily living [18]); quality of life (using the EuroQoL EQ-5D questionnaire [19]); extended activities of daily living (using the Nottingham Extended Activities of Daily Living (NEADL) index [20]).

Participants who die before the assessment point will not have data for the NIHSS, Barthel index, EuroQol EQ-5D, or NEADL index. This could bias results in favor of the treatment arm with higher mortality. Death will therefore be included in the analysis of the NIHSS, EuroQol EQ-5D, and NEADL index as the worst outcome on the scale [21].

For numerical outcomes, means and standard deviations or medians and interquartile ranges will be reported, as appropriate. Unadjusted analyses will use an unrelated *t* test, with the mean difference between treatments and appropriate confidence interval reported. In the event of major deviations from the assumptions of the *t* test, an appropriate alternative analysis will be used. The adjusted analysis will use analysis of covariance methods, with the covariates specified earlier included in the analysis.

For dichotomous outcomes, percentages will be compared across the treatment comparisons using a *χ*-squared or Fisher exact test as appropriate for the unadjusted analysis. The adjusted analysis of dichotomous outcomes will use binary logistic regression, using the covariates listed earlier. Odds ratios and confidence intervals will be reported. The number needed to be treated will also be calculated [22].

For ordinal secondary outcomes, the analyses described for the mRS will be applied.

### Data at 6 and 12 months

The longer-term follow-up data at 6 and 12 months will be analyzed at each time point using the same methods described earlier. In addition, analyses will be performed across 3-, 6- and 12-month time points using a longitudinal repeated measures analysis, such as linear mixed models [23].

The treatment effect will initially be assumed to be constant over time; further analyses may be carried out to investigate the effects of including time and a treatment × time interaction in the models.

Mortality will be analyzed using log-rank methods (unadjusted analysis) with Kaplan-Meier plots presented. The adjusted analysis will use Cox regression methods, including the covariates listed previously. In the covariates, the prognostic index for 30-day survival will replace that for independence at 6 months. The proportional hazards assumption associated with the Cox regression model will be tested via Schoenfeld residuals (this assumption was found to be tenable in the analysis of the 6-month survival in the pilot study [24]). Hazard ratios and 99% confidence intervals will be reported for both unadjusted and adjusted analyses.

### Planned subgroup analyses

These will be performed in respect of the primary outcome measure only, based on a risk-stratification approach [25]. The subgroups comprise: NIHSS score at baseline as indicator of stroke severity (0 to 4, 5 to 9, 10 to 14, 15 to 20, >20); baseline% oxygen saturation (<92, 92 to 93.9, 94 to 94.9, 95 to 97, >97); treatment with O_2_ prior to randomization (yes or no); time in hours since onset of stroke (≤3, >3 to 6, >6 to 12, >12 to 24, >24); type of stroke (hemorrhage or infarct); Glasgow Coma Scale motor score plus eye score (<10, 10); age (<50, 50 to 80, >80); history of chronic obstructive airways disease or asthma (yes or no); history of heart failure (yes or no); thrombolysis (yes or no); baseline SSV risk score for independence at 6 months (≤0.1, >0.1 and ≤0.35, >0.35 and ≤0.7, >0.7).

These subgroup effects will be analyzed by means of an interaction term [26]; however, pairwise hypothesis tests between the levels of the subgroup factor will not be performed, owing to the probably low level of statistical power. Subgroup-specific estimates will be reported descriptively with 95% confidence intervals and displayed graphically in a forest plot, and will be interpreted with caution (especially in respect of any subgroups with low numbers).

### Exploratory analyses

Exploratory analyses will be conducted using data collected at 7 days. These will include details of the stroke diagnosis and imaging (for example, imaging results, final diagnosis, stroke syndrome [27], etiological classification [28], indicators of compliance with the trial treatment, oxygen saturation during the intervention), and clinical data that might indicate stress induced by the intervention, for example, sedative use, the number of participants whose highest heart rate was >100 beats per minute, whose highest systolic blood pressure was >200 mmHg, or whose highest diastolic blood pressure was >100 mmHg during the intervention, or who developed infections (antibiotic use during the first 7 days).

In addition, we will report data on symptoms that were highlighted as important for their quality of life by stroke patients and their carers [29], but not sufficiently covered in the validated tools used for the assessment of the primary and secondary outcomes. These are: the number of patients who reported their memory, eyesight, and sleep as being ‘as good as before the stroke’ , and the number who reported that they had no significant speech problems (no problems or some problems but not interfering significantly with conversation). These outcomes were adapted from the ‘simple questions’ described by Lindley *et al.*[30], but are not validated.

For these exploratory analyses, data will be tabulated across the treatment comparisons at each time point, but will not be subjected to formal statistical testing.

### Sensitivity analyses

The following sensitivity analyses will be performed.

Two comparative analyses with the observed case analysis will be performed to allow for missing data. First, a multiple imputation method, using at least ten imputed datasets, will be used. These imputations will be based on specified baseline covariates (age, sex, treatment group, oxygen saturation, SSV risk score, NIHSS score) and values of the outcome variable concerned at other time points. If necessary, missing data on baseline covariates used in the multiple imputation algorithm will be estimated [31]. Second, two additional imputations will be conducted to allow for the possibility that data is missing not at random, and missing values are (i) better or (ii) worse than would otherwise be expected.

Two comparative analyses with the intention-to-treat analysis will be performed [32]. First, a per-protocol ‘adherers only’ analysis will be conducted, where only patients who complied with treatment are analyzed. Second, a per-protocol ‘as treated’ analysis (if feasible) will be carried out, where patients are classified with respect to the intervention that they ultimately received rather than the intervention to which they were randomized.

Additionally, in the event that the proportional odds assumption for analysis of the main outcome does not hold, an appropriate alternative method will be investigated, such as a sliding dichotomy analysis [33].

### Serious adverse events

The proportion of patients who experience at least one serious adverse event will be analyzed as a categorical variable (with patients classified as either having experienced at least one serious adverse event, or not) using a *χ*-squared or Fisher’s exact test (as appropriate). If appropriate, a more complex model of serious adverse event occurrences will be constructed utilizing adverse events as count variables. Possible models for this analysis include Poisson regression and negative binomial regression. An analysis of subgroups of serious adverse events will be performed separately, but in an identical manner to the overall adverse events analyses.

## Health economic analyses

As indicated in the trial protocol [13], there will also be an economic analysis. The details of this analysis are documented separately.

## Status

The final version of this statistical analysis plan was approved by the trial steering committee on 4 March 2014.

## Abbreviations

mRS: modified Rankin scale; NEADL: Nottingham Extended Activities of Daily Living; NIHSS: National Institutes of Health Stroke Scale; SO_2_S: Stroke Oxygen Study; SSV: six simple variables.

## Competing interests

The authors declare that they have no competing interests.

## Authors’ contributions

JS: conception and design of work, manuscript writing and final approval of manuscript. RG: conception and design of work, critical revision and final approval of manuscript. TN and NI: design of work, critical revision, and final approval of manuscript. AH: design of work, manuscript writing, critical revision and final approval of manuscript. CR: conception and design of work, manuscript writing, critical revision, and final approval of manuscript. All authors read and approved the final manuscript.
